# Inadequate iodine status among women of childbearing age in Northern Norway: a cross-sectional study

**DOI:** 10.29219/fnr.v69.10802

**Published:** 2025-02-26

**Authors:** Maren Johnsen, Guri Skeie, Tonje Braaten, Marianne Hope Abel, Sandra Huber, Marian Kjellevold, Elin Evensen, Margaret Rayman, Solrunn Hansen

**Affiliations:** 1 Department of Health and Care Sciences, Faculty of Health Sciences, UiT The Arctic University of Norway, Tromsø, Norway; 2 Department of Community Medicine, Faculty of Health Sciences, UiT The Arctic University of Norway, Tromsø, Norway; 3 Department of Physical Health and Ageing, Norwegian Institute of Public Health, Oslo, Norway; 4 Department of Laboratory Medicine, University Hospital of North Norway, Tromsø, Norway; 5 Institute of Marine Research (IMR), Bergen, Norway; 6 Department of Nutritional Sciences, Faculty of Health and Medical Sciences, University of Surrey, Guildford, United Kingdom

**Keywords:** iodine status, iodine intake, pregnancy, women, nutrition

## Abstract

**Background:**

Iodine is crucial for thyroid hormones, normal metabolism, growth and development in the foetal period. Low iodine status in women of childbearing age is particularly worrying since iodine deficiency continues into pregnancy.

**Objective:**

This study aimed to measure iodine status in non-pregnant and pregnant women in Northern Norway and investigate group differences and determinants of urine iodine concentrations (UICs) based on dietary factors and participants’ knowledge about iodine.

**Methods:**

This cross-sectional study included pregnant (*n* = 131) and non-pregnant (*n* = 493) women from the Northern Norway Mother-and-Child Contaminant Cohort Study 2 study (2017–2021) and the Fit Futures 3 study (2020–2021). UIC was measured in spot urine, and dietary iodine intake was calculated from food frequency questionnaires. Group differences in median UIC were explored using non-parametric tests. Associations between independent variables and median UIC were estimated through quantile regression, adjusting for relevant covariates.

**Results:**

Median UIC was 91 μg/L in non-pregnant and 134 μg/L in pregnant women, thus below the World Health Organization definition of insufficient iodine status of < 100 μg/L and 150 μg/L, respectively. Dairy products and lean fish were the most important dietary iodine sources, but the median estimated intake did not reach the recommended intake. Taking iodine supplements was the strongest determinant of UIC in both groups (*P* < 0.01), and users had adequate iodine status at a group level. A high proportion of the non-pregnant women (84%) were not taking iodine supplements. Poor knowledge about iodine in the participant groups was observed but was not associated with UIC.

**Conclusion:**

Pregnant and non-pregnant women not using iodine supplements had inadequate iodine status and insufficient iodine intake. Supplement use or interventions at the societal level are essential to ensure adequate status in these vulnerable groups.

## Popular scientific summary

- New data on iodine status on women in childbearing age from the region Northern Norway in Norway are needed in the absence of a national monitoring and fortification programme.
- This is the first iodine study from Northern Norway After an increased national focus on iodine deficiency in vulnerable groups.
- Results indicate that non-pregnant and pregnant women not using iodine supplements had inadequate iodine status and insufficient iodine intake.

Iodine is an essential trace element crucial for the synthesis of thyroid hormones and normal metabolism, and growth and development in humans. As iodine requirements increase during pregnancy, an adequate intake is important not only for pregnant women but also for women of childbearing age to prepare them for potential pregnancies (1–3). During the foetal stages, especially during the first trimester and neonatal period, iodine is crucial for foetal brain development and for preventing pregnancy loss, neonatal hypothyroidism and cretinism (4). If a woman enters pregnancy with depleted iodine levels, it can be critical for the foetus, and even a mild-to-moderate deficiency may affect the neurocognitive development of the child, resulting in poorer fine motor skills and reduced intelligence quotient (5). Therefore, young women of childbearing age need special consideration in relation to iodine deficiency since a deficiency may continue into pregnancy and affect the developing child (2, 6).

Despite progress in iodine fortification policies and in periodic monitoring of iodine status worldwide, a systematic review from 2022 of 61 studies from 2008 to 2021 estimated an overall prevalence of insufficient iodine intake of 53% among pregnant women (7). In Norway, mild-to-moderate iodine deficiency related to pregnancy has been documented since 2013 (8), with increased national awareness since 2016, and the recognition that women of childbearing age constitute a vulnerable population (9). In the Norwegian MoBa (The Norwegian Mother, Father and Child Cohort Study) study from 1998 to 2008, the median iodine intake from food in pregnant women was 121 μg/day (*n* = 78,317), while the median urine iodine concentration (UIC) was 69 μg/L (*n* = 2,795), both below the World Health Organization (WHO) recommended cut-off values of 250 μg iodine per day and median UIC ≥ 150 μg/L for pregnant women. Findings from Northern Norway, that is, the Northern Norway Mother-and-Child Contaminant Cohort Study (MISA) 1 study from 2007 to 2009, revealed a median UIC of 84 μg/L, which indicates mild-to-moderate iodine deficiency at group level (10). Similar results were documented in the national Little in Norway (LiN) study from 2011 to 2014 (*n* = 954, median UIC: 85 μg/L) (11). More recently, regional studies in parts of Southern Norway conducted since 2017 have also detected iodine deficiency in pregnant women (12, 13). Furthermore, numerous studies from Western countries have examined iodine status in young schoolgirls and adolescents, with a majority, including Norwegian research, revealing mild-to-moderate deficiency (14, 15). In contrast, research focusing on women in their twenties is scarce. However, the few studies conducted in Norway and other European countries suggest that mild-to-moderate iodine deficiencies are also prevalent in this age group (12, 16, 17, 18).

Previous studies have shown that there is low awareness of iodine among women of childbearing age in Norway, both regarding the high risk of deficiency and the importance of iodine (12, 19). Despite this association, there remains a notable lack of research focused on exploring this field in this population (20). Since the 1950s, iodine has been added to livestock feed in Norway, which has increased the iodine content of milk. As a result, cow’s milk and lean fish that naturally contains iodine are the primary iodine sources in the Norwegian diet. This approach differs from that of other Nordic and European countries, where iodised salt programmes play a significant role in iodine intake (9, 21, 22). It is a cause for concern that the consumption of fish and dairy products has decreased in Norway and the other Nordic countries in recent decades (22, 23).

Since 2016, the Norwegian National Council for Nutrition has recommended a mandatory iodine fortification programme of table salt and of salt used in manufactured bread and pastries because of the iodine deficiency among women of childbearing age (9), but this has not yet been implemented.

Due to repeated indications of mild-to-moderate deficiency among Norwegian women of childbearing age, in addition to previous results from Northern Norway, new data from this region are needed in the absence of a national monitoring and fortification programme. The aim of the present study was to measure iodine status in pregnant and non-pregnant women in Northern Norway, to investigate their knowledge of iodine, dietary intake and supplement use and to examine potential differences in iodine status by dietary factors.

## Materials and methods

### Study design

The present study utilises data from two different studies, the MISA 2 study (*n* = 244) and the Fit Futures 3 study (*n* = 380), both administered by UiT The Arctic University of Norway. Recruitment and sampling for both studies took place in Northern Norway. Relevant to this iodine study, the participants provided spot urine samples and answered questionnaires on diet and background information.

The MISA cohort study, initiated in 2007, had the aim to investigate concentrations of persistent toxic substances in women and children related to pregnancy and breastfeeding in Northern Norway (24). As an extension, MISA 2 was started in 2017. It focuses on diet, lifestyle factors and persistent toxic elements related to reproductive health and includes mother-child dyads during breastfeeding periods. Non-pregnant and pregnant women were eligible for the present study. From the region of Bodø, 112 pregnant women who attended a public or private obstetric clinic were recruited from June 2019 to June 2021. Examinations were performed by a research assistant at the private clinic. Furthermore, through e-mail invitations distributed by selected universities, 140 non-pregnant female healthcare students aged 30 or below were recruited at two campuses, namely, Nord University, campus Bodø, and UiT The Arctic University of Norway, campus Hammerfest. Of these, three informants withdrew their consent. Temporary research stations were established at Nord University for 4 weeks and 3 days in November 2017, January 2018 and October 2019, and at UiT for 7 days in September 2017. Trained personnel were responsible for the data collection. In the present study, five participants were excluded due to missing urine samples or not having been analysed for iodine. Thus, the final MISA 2 study group included 135 non-pregnant and 109 pregnant women.

The Fit Futures study (25) is a population-based cohort study with three repeated health surveys among adolescents, now young adults, mainly from Tromsø, the largest city in Northern Norway. The third wave of data collection in the Fit Futures study (hereafter called FF3) took place from March 2021 to March 2022. In FF3, a total of 705 (55% women) from the original cohort from Fit Futures 1 and 2 participated, giving a participation rate of 61%. The data collection took place at the Clinical Research Unit of the University Hospital of North Norway (UNN), Tromsø. In the present study, we included 22 pregnant and 358 non-pregnant women from FF3.

### Questionnaires

The same day as the urine sample was collected, the MISA and FF3 participants answered a short questionnaire that addressed frequency in the past week of intake of foods high in iodine, such as dairy products, which include milk (four categories), yoghurt, sour cream and cheese (five categories), fish and seafood (eight categories), fish sandwich spread (four categories) and eggs. Response options ranged from no intake to four or more times per day, but four or more times per week for fish. These responses were our main source of dietary iodine information.

In addition, participants in MISA 2 were asked to answer an extensive online questionnaire within 14 days of inclusion, with a response rate of 78%. Questionnaires on habitual diet in the preceding year were, with some additions, retrieved from the MISA 1 study’s four-page food frequency questionnaire (FFQ) and originated from the validated FFQ in the Norwegian Women and Cancer study (26). The dietary questions in the short questionnaire have not been validated but are based on categories from the four-page FFQ with the addition of some new dairy products, selected and organised according to iodine content as described below. For both dietary questionnaires, nutrient calculations were performed. Based on the Norwegian Weight and Measurement Table (27), the consumption of each food item is expressed in grams per day, and the daily intake of energy and the nutrient iodine was calculated using values from the Norwegian Food Composition database (28).

Both MISA and FF3 inquired about iodine supplements (‘Do you take iodine supplements?’) and product names. In MISA 2, participants also answered the question, ‘During the last week, did you take iodine supplements?’ Iodine supplements were double-checked, and if multivitamins were taken, product information was checked for iodine content. Iodine from supplements was not included in the intake estimations.

Other questionnaire information retrieved for the present study includes demography and lifestyle factors such as age (years), body mass index (BMI, kg/m^2^), education (3 years of university or higher), smoking and use of snuff (a type of smokeless tobacco) (yes/no), parity (women with no previous pregnancies/women with one or more previous pregnancies), pregnancy week, thyroid diseases (yes/no) and thyroid medication (yes/no). From the MISA study only, knowledge about iodine was obtained: its importance, its function, national recommendations, iodine sources and the extent to which the participant had received information from healthcare personnel about the importance of an adequate iodine status. A detailed description of these questions adapted from other studies (29–31) can be found in our previous publication (32).

### Analytical methodology

#### Urine sampling and storage

Spot urine from each participant was collected at inclusion. In the MISA study, urine was collected in urine containers, 25 mL Sarsted polypropylene (PP) (# 63.9922.252), and transferred to Sarsted PP 10 mL tubes (# 62.9924.283). In FF3, the urine was collected in standard urine containers (100 mL), aliquoted and transferred to 10 mL tubes. The tubes were immediately stored at −20°C until transport to the UNN Environmental Pollutant Laboratory for further storage at −35°C.

#### Measurement of iodine status

WHO recommends assessing the median UIC to determine a population’s iodine status. The UIC is a good indicator of recent iodine intake because > 90% of iodine intake is eliminated in the urine (WHO). Even though the UIC has a substantial day-to-day variation, it is our best measurement at the population level (3). The WHO criteria for the evaluation of iodine status were used in this study (3). The WHO reference values reflecting sufficient iodine status are UIC ≥ 150 μg/L for pregnant women and ≥ 100 μg/L for non-pregnant women. Furthermore, the WHO defines more than 20% of values < 50 μg/L as an additional indication of mild-to-moderate deficiency (3).

In order to adjust for urine dilution effects in spot urine samples (33), creatinine adjustment was performed as follows: urinary iodine concentration [μg/L]/creatinine concentration [g/L] = μg iodine per g creatinine.

#### Chemical analysis

Analyses of UIC were performed at the UNN Environmental Pollution Laboratory using inductively coupled plasma mass spectrometry measurements. Sample preparation and instrumental analysis were performed as follows: 200 μL of the MISA and 100 μL of the FF3 urine samples were diluted 1:20 with an alkaline reagent consisting of 0.08% Triton X-100 (Sigma/Merck KGaA, Darmstadt, Germany), 0.25 μg/L gold standard (Au; Inorganic Ventures, Christiansburg, VA, USA), 10% trace-SELECT 2-propanol (Honeywell/Riedel de Haen, Bucharest, Romania) and 10% suprapure ammonia (Merck KGaA, Darmstadt, Germany) in ultrapure water dispensed from a Milli-Q Advantage A10 ultrapure water purification system (Merck KGaA, Darmstadt, Germany). Matrix-matched calibration standards and study samples, procedure blank samples and quality control samples were prepared on a Tecan Freedom Evo 200 liquid handler (Männedorf, Switzerland) in 48-well plate format. Instrumental analysis was performed using a Perkin Elmer NexION 300D ICP-MS instrument (Perkin Elmer, Waltham, MA, USA) coupled to an ESI-Fast SC2DX autosampler (Elemental Scientific Inc., Omaha, NE, USA). Kinetic energy discrimination mode with 4.8 mL helium flow was used for mass spectrometry analysis. A four-point calibration curve was used for quantification with linear regression through zero, together with blank subtraction after internal standard correction (rhodium; Inorganic Ventures, Christiansburg, VA, USA). Method detection limits (MDLs) were calculated as three times the standard deviation (SD) of the concentrations of the procedure blank samples analysed during the analysis period of the MISA 2 and FF3 urinary samples. MDLs were calculated as 0.182 μg/L in MISA 2 samples and 2.098 μg/L FF3 samples.

Creatinine measurements were also performed at the Department of Laboratory Medicine, UNN, on a Cobas 8000 c702 analytical unit (Roche Diagnostics, Mannheim, Germany) with the enzymatic method developed by Roche. The laboratory is ISO 15189 accredited (34).

### Statistical analyses

Statistical analyses were performed in STATA (version 16.0; Stata Corp., College Station, TX). Descriptive data and characteristics are presented as frequencies and percentages, means and SDs, and/or median, minimum and maximum values. The Mann-Whitney U test was used to compare median UIC between the study groups. We computed Spearman’s correlation coefficient between iodine intake and UIC. Multivariable quantile regression models were used to model differences in median UIC by relevant dietary factors containing high amounts of iodine (iodine supplements, lean fish and dairy products). Quantile regression was used because the distribution of the outcome measure was highly skewed, and in addition, the median is used to define cut-off values for iodine deficiency (WHO). There were no extreme values of UIC > 1,000 μg/L. Results are reported as coefficients with corresponding 95% confidence intervals (CIs) and *P*-values. Robust standard errors were used to account for heteroskedasticity and misspecification. Relevant background variables were tested in the model and were discarded for further modelling if the *P*-value was > 0.05 (Supplementary Table 2). A *P-*value < 0.05 was considered statistically significant.

### Ethics

This study was approved by the Northern Norway Regional Committee for Medical and Health Research Ethics (REC North-#717079). All the participants received written information about the study and signed up voluntarily, and this enrolment requires written consent from the participant. This study was conducted in accordance with the Helsinki Declaration.

Before the research started, two young women studied and provided feedback on the questionnaire to ensure service user involvement. Furthermore, two non-pregnant students and two pregnant women contributed their perspectives on the results before the discussion section of this article was written.

## Results

### Participant characteristics

Background characteristics are presented in Table 1. The mean age of the non-pregnant women was 24 years, and 56% had a BMI in the normal range below 25 kg/m^2^. The pregnant women had a mean age of 28 years, and 51% had a normal pre-pregnant BMI (Table 1). Eight percent of the participants reported current smoking, and 37% reported using snuff (a practice of placing finely ground tobacco under the upper lip). None of the pregnant women reported smoking, but 5.3% stated that they used snuff. The use of thyroid medicines was limited to nine participants among the non-pregnant women and one in the pregnant group. Most of the non-pregnant women in MISA 2 were students, while FF3 participants were more diverse as shown in Supplementary Table 1, which includes background characteristics in MISA 2 and FF3. In the pregnant group, the majority (73%) had higher education.

**Table 1:** Participant characteristics^1^ for the MISA 2 study and the Fit Futures 3 study

	Non-pregnant, *n* = 493	Pregnant, *n* = 131
	*n* (%)	*n* (%)
Age (years), mean (± SD)	24 ± 1.8	28 ± 2.7
min-max	19–36	20–46
BMI (kg/m^2^), mean^2^	25.8	26.4
≤ 25	273 (55.6)	51 (51)
> 25	218 (44.4)	49 (49)
Pregnancy week, mean (± SD)	-	15 ± 7.3
Parous		
Primi	-	59 (45)
Multi	-	72 (55)
Smoking status (yes)^3^	36 (7.6)	0 (0)
Snuff status (yes)^3^	178 (37.3)	7 (5.3)
Thyroid disease^4^	9 (1.8)	1 (0.8)
Higher education^5^	238 (48.3)	72 (73.4)

1 Missing: Age: 0/1, BMI 2/31, Preg.week -/0, parous -/0, smoking 23/0, snuff 16/0, thyroid disease 5/3, higher education 39/33. (Reflects undelivered questionaires).

2 Pre-pregnant for those pregnant.

3 The women were asked “During the last week have you..”, “Sometimes” or “Daily”.

4 Using medication for the condition.

5 Higher education: University, minimum bachelor’s degree. All non-pregnant women in MISA were current bachelor’s degree students.

### Urine iodine concentrations and iodine intake

The non-pregnant women had a median UIC of 91 μg/L (CI 84–101), while the pregnant women had a median UIC of 134 μg/L (CI 122–152) (Table 2). Among the non-pregnant women, 21% had a UIC below 50 μg/L. In contrast, only 6.8% of the pregnant women had a UIC below 50 μg/L. UIC adjusted for creatinine (UIC/UCr) was 61 μg/g and 113 μg/g for non-pregnant and pregnant women, respectively (Table 2).

**Table 2:** Urinary iodine concentration (UIC, μg/L) and iodine intake among participants

	Non-pregnant	Pregnant	*p*-value^1^
	*n* = 493	*n* = 131	
**Urinary iodine concentration**			
median UIC (μg/L)	91	134	**< 0.001**
95% CI	84–101	122–152	
< 50 ug/L^2^	102 (20.7)	0	
**Iodine:creatinine (μg/g)**			
median	61	113	**< 0.001**
95% Cl	57–64	84–132	
**UIC (μg/L) by supplement use**			
Yes	120	158	
No	88	92	
**Estimated iodine intake during the last 7 days (μg/day)**			
median	124	147	0.02
min-max	0–604	0–490	
**Reported intake of iodine food sources the last 7 days (g/day)**			
Dairy products^3^	200	240	**0.01**
min-max	0–1015	0–2570	
Lean fish^4^	29	34	**0.04**
min-max	0–186	0–115	
Oily fish^5^	21	21	0.90
min-max	0–158	0–67	
Eggs	19	19	0.27
min-max	0–135	0–135	

1 Mann-Whitney test between non-pregnant and pregnant women.

2 WHO recommendations: Not more than 20% of sample should be below < 50 μg/L.

3 Dairy products: All dairy products including milk and cheese.

4 Lean fish: cod, haddock, saithe or other fish.

5 Fat fish: salmon, mackerel or trout.

Examination of consumption of foods rich in iodine during the last 7 days revealed a significant difference between the estimated median dietary intake of iodine in non-pregnant (124 μg/day) and pregnant (147 μg/day) women. Pregnant women consumed significantly more dairy products (*P* = 0.012) and fish (*P* = 0.047) than non-pregnant women (Table 2). In both groups, dairy products were the most frequently consumed foods rich in iodine, with a considerably lower intake of fish and eggs (Table 2). Nevertheless, around 30% of the pregnant women and 40% of the non-pregnant women reported no consumption of dairy products during the week before the urine sampling. More non-pregnant women also reported no intake of lean fish (53%) than pregnant women (42%) (Table 3). The UIC was significantly higher in participants who consumed dairy products than those reporting no intake of dairy products (*P* < 0.05, Table 3).

**Table 3:** Observed differences in median urinary iodine concentrations (UIC, μg/L) by supplement use, consumption and recommendations among participants^1^

	Non-pregnant, *n* = 493		*p*-value^2^	Pregnant, *n* = 131		*p*-value^2^
	*n* (%)	median UIC (μg/L)		*n* (%)	median UIC (μg/L)	
**Iodine supplement**						
Yes	78 (15.9)	120	**< 0.002**	77 (60)	158	**< 0.001**
No	414 (84.1)	88		51 (40)	92	
**Iodine supplement before pregnancy^3^**						
Yes	-	-	-	17 (16.5)	146	0.16
No	-	-		82 (79.6)	104	
**Folic acid supplement before pregnancy^3^**						
Yes	-	-	-	54 (50.5)	134	0.50
No	-	-		53 (49.5)	139	
**Consumption of dairy products the last week**						
No intake at all	188 (40.2)	91	**0.05**	38 (29.9)	117	**0.02**
Intake of dairy^4^	280 (59.8)	95		89 (70.1)	145	
**Consumption of lean fish the last week**						
No intake at all	262 (53)	91	0.60	55 (41.9)	132	0.30
Intake of lean fish^4^	231 (47)	97		76 (58.1)	142	
**Achieved recommended iodine intake, WHO^5^**						
Yes	229 (46.5)	151	**< 0.001**	55 (41.9)	236	**< 0.001**
No	264 (53.5)	59		76 (58.1)	91	

1 Missing: 3/1, -/10, -/2, 4/25, 0/20, 0/0.

2 Mann-Whitney test between yes/no^,^ no intake/intake.

3 Only MISA 2 participants (*n* = 109) were asked this question.

4 Varied consumption of lean fish or dairy products, from once a week to every day during the last week.

5 WHO recommendations: 150 μg for non-pregnant, 200 μg for pregnant.

Regarding the use of iodine supplements during the last week, 16% of the non-pregnant women reported use compared to 60% of the pregnant women (Table 3). Median UIC differed significantly depending on whether participants were taking iodine supplements. In the pregnant group, UIC was 158 μg/L in the iodine supplement users, compared to 92 μg/L in the non-users. A clear difference was also seen in the non-pregnant group, with UICs of 120 μg/L and 88 μg/L (Table 3 and Supplementary Figures 1 and 2). Additionally, restricted to MISA 2 (*n* = 109), 17% of the pregnant women reported having used iodine supplements prior to becoming pregnant, while 50% reported having taken folic acid (Table 3). A weak correlation (*r* = 0.12, non-pregnant, *r* = 0.23, pregnant) was found between participants’ spot UIC and daily iodine intake from food products during the last week.

### Knowledge about iodine among participants in the MISA 2 study

According to Table 2 and restricted to the MISA 2 study, a substantial majority of the participants were aware of the essential role of iodine in nutrition. However, only 27% of pregnant participants assumed that their iodine intake met dietary needs, compared to 50% of non-pregnant women. Half of the pregnant participants recognised inadequate iodine intake as a prevalent problem in Norway, whereas only one in four non-pregnant participants shared this view. Furthermore, 28% of pregnant women and 10% of the young, non-pregnant women reported having received iodine-related information from healthcare professionals. In terms of identifying iodine-rich foods, a higher proportion of pregnant women correctly identified dairy and fish products as good sources, while non-pregnant women showed poorer knowledge. Both groups displayed limited knowledge of eggs as a source of iodine. Additionally, both pregnant and non-pregnant women exhibited little knowledge of the importance of iodine for their own metabolism and normal foetal growth and development during pregnancy and lactation. None of the questions in Table 4 were significantly associated with UIC (Table 5).

**Table 4:** Urinary iodine concentration (UIC, μg/L) stratified on iodine information received from health care personnel and iodine knowledge among MISA 2 participants^1, 2^

	Non-pregnant *n* = 135			Pregnant *n* = 109		
	*n* (%)	median UIC (μg/L)	*p*-value^3^	*n* (%)	median UIC (μg/L)	*p*-value^3^
**Received information about iodine from health care personnel**						
Yes	11 (10.2)	149	0.15	21 (28.4)	130	0.74
No	69 (63.8)	108		41 (55.4)	147	
Don’t remember	40 (26.0)	111		12 (16.2)		
**Believe they get enough iodine through diet**						
Agree	54 (50)	107	0.61	20 (27)	142	0.37
Disagree	14 (13)	124		18 (24.3)	110	
Don’t know	40 (37)	94		36 (48.7)	158	
**Knowlege about iodine**						
Iodine as an essential nutrient						
Yes	78 (72.9)	103	0.85	56 (75.7)	139	0.83
No	13 (12.1)	108		12 (16.2)	132	
Don’t remember	16 (15.0)	80		6 (8.1)	202	
Iodine status among pregnant/lactating women in Norway						
*A low intake is a problem*	27 (25.2)	111	0.37	37 (50)	134	0.90
*A low intake is not a problem*	13 (12.1)	97		2 (2.7)	112	
*Don’t know*	67 (62.7)	106		35 (47.3)	147	
The most important source of iodine through diet^4^						
*Dairy products*	35 (31.2)	122	0.19	50 (67.6)	133	0.57
*Fish and seafood*	45 (40.1)	109	0.26	42 (57.5)	132	0.48
*Eggs*	30 (26.8)	156	0.07	17 (25.7)	152	0.37
*Iodised salt*	41 (36.6)	86	0.30	23 (35.5)	170	0.14
*Supplement*	8 (7.14)	158	**0.05**	11 (18.0)	149	0.88
What is iodine important for^4^						
*Normal foetal growth and development during pregnancy*	22 (19.6)	93	0.96	23 (36.5)	156	0.52
*Normal growth and development in children*	23 (20.5)	127	0.19	27 (40.9)	146	0.86
*To maintain a normal metabolism*	39 (34.8)	113	0.27	17 (27.4)	152	0.42
*Don’t know*	41 (36.6)	91	0.57	21 (34.4)	144	0.98

1 Questions only asked of MISA2 participants, not Fit Futures participants.

2 Missing: 35/28 (Reflects undelivered questionnaires).

3 Mann-Whitney test between yes and no. Don’t remember/don’t know not included in the test.

4 Possible to answer with one or more alternatives.

**Table 5:** Estimated differences in median urinary iodine concentration (μg/L) by dietary factors and supplement use^1^

	Median UIC (μg/L)			
	Non-pregnant, *n* = 493		Pregnant, *n* = 131	
	Model 0^2^	Model 1^3^	Model 0^2^	Model 1^3^
	Beta (95% CI)	Beta (95% CI)	Beta (95% CI)	Beta (95% CI)
**Iodine supplement**				
No	Ref.	Ref.	Ref.	Ref.
Yes	31 (10, 52)	30 (10, 49)	57 (16, 98)	70 (32, 107)
	***p* = 0.005**	***p* = 0.003**	***p* = 0.007**	***p* < 0.001**
Lean fish, per portion(150 g) per week^4^	0.1 (-2, 3)	0.7 (-4, 2.2)	4 (-6, 13)	2.9 (-6, 12)
	*p* = 0.94	*p* = 0.65	*p* = 0.5	*p* = 0.53
Oliy fish, per portion(150 g) per week^4^	6 (1, 11)	4 (-0.4, 9)	16 (7, 40)	20 (5, 35)
	***p* = 0.015**	***p*** = 0.07	***p* = 0.005**	***p* = 0.01**
Dairy products, glasses(1.5 dl) per day^4, 5^	22 (13, 30)	23 (15, 31)	15 (2, 27)	18 (6, 30)
	***p* < 0.001**	***p* < 0.001**	***p* = 0.02**	***p* < 0.001**

1 Differences in median urinary iodine are estimated based on quantile regression.

2 Model 0: Crude- not adjusted for covariates.

3 Model 1: Adjusted for all covariates listed in table.

4 Reported last week.

5 Also includes cheese.

### Determinants of urinary iodine concentration

The use of iodine supplements and the consumption of dairy products and oily fish were positively associated with median UIC (Table 5). The use of iodine supplements was associated with a 70 μg/L higher median UIC in pregnant women (*P* < 0.001) and a 30 μg/L higher median in non-pregnant women (*P* = 0.003). The median UIC was estimated to be 18–23 μg/L higher for each extra glass of dairy products consumed (*P* < 0.001). Each portion of oily fish last week was associated with a 20 μg/L higher median UIC increase in pregnant women (*P* < 0.01) but was not significantly associated with UIC in the non-pregnant women. In both groups, no association was seen between UIC and lean fish (Table 4). Among the MISA 2 participants, we were able to repeat the analyses based on annual estimations of dietary factors and UIC. This gave us similar results; however, oily fish was not associated with elevated UIC before or after adjusting the model in the pregnant group (Supplementary Table 3).

Furthermore, there was no association between UIC and participants’ background characteristics, knowledge questions, or information received about iodine (Supplementary Table 2).

## Discussion

This is the first iodine study from Northern Norway in women of childbearing age since the increased national focus on iodine deficiency in vulnerable groups. Using the WHO recommendations as the benchmark, our study has revealed insufficient iodine status in both non-pregnant and pregnant women. Our findings suggest that dietary iodine intake from food sources is insufficient. However, the use of iodine supplements is linked to higher median UIC, indicating iodine sufficiency at group level in supplement users. Nevertheless, a significant number of individuals, particularly in the non-pregnant group, did not take such supplements regularly. Coupled with a general trend of low consumption of foods rich in iodine was widespread poor knowledge about iodine, and the critical role this trace element plays for the body and the foetus.

Our results are of concern since they reflect insufficient iodine levels in young women of childbearing age. The latest study among non-pregnant women from Norway, with data collected between 2017 and 2021, also suggests an inadequate median UIC of 75 μg/L (29) and a median iodine intake of 117 μg/day based on 24-h recalls (35). Establishing adequate iodine status is a gradual process that can take from a few months to 2 years (36, 37). Women of childbearing age face the risk of iodine deficiency during pregnancy, which can have detrimental consequences. An uncertain iodine status at conception and in the initial weeks leading up to a confirmed positive pregnancy test further increases the vulnerability of this group (22).

Our results give indications of iodine deficiency in pregnant women. Considering the increased iodine requirements during pregnancy, the pregnant participants’ median UIC levels of 134 μg/L still fell below the levels recommended by the WHO (3), and the pregnant women who did not take an iodine supplement had a median UIC level of only 92 μg/L. Despite some improvement in recent years, this result confirms the continued evidence of insufficient iodine status in the Norwegian pregnant population (11, 29, 38). The latest study on pregnant women from Western Norway conducted in 2021 reported a median UIC of 94 μg/L, which is considerably lower than in our pregnant group, but comparable to our pregnant non-supplement users (92 μg/L) (13). Similarly, recent studies from other Nordic countries, Finland and Iceland, showed a median UIC in pregnant women of 117 μg/L and 89 μg/L, respectively (39, 40). Similar to our results, these studies showed that the use of iodine supplements was associated with a higher median UIC (39). Furthermore, the UIC level among non-supplement users in Iceland, 87 μg/L (40), was comparable to our study, which showed a similar median, namely, 88 μg/L. Nevertheless, a more accurate comparison might be drawn by comparing our results with previous findings from our MISA 1 study conducted in 2007–2009, which revealed a median UIC of 75 μg/L (10). Thus, even though the median UIC in the present study is still below sufficient iodine status as defined by the WHO, our present results are encouraging. It is pertinent to note that the Nordic Nutrition Recommendations (NNR) suggest that a median UIC concentration of ≥ 105 μg/L is indicative of meeting the recommended daily iodine intake for pregnant women of 175 μg. This recommendation is notably lower than the WHO guideline, which advocates a daily iodine intake of 250 μg in pregnancy (3).

Regardless of pregnancy status, our findings demonstrated that dietary intake does not cover the daily requirement for iodine as recommended by WHO and the NNR (3, 9). Comparing our results with the MISA 1 study (10) reveals an increase in iodine intake from 72 to 147 μg/day in pregnant women. However, the intake of key iodine-rich food items such as dairy products was still notably low. Dairy products were the strongest contributor to iodine-rich food items in our study. This finding corroborates with reports that identify dairy products as the predominant source of iodine in the contemporary Norwegian diet (21).

The median daily consumption of merely ~30 g of lean fish is slightly lower than the Norwegian dietary recommendation of 250–450 g of all types of fish per week (41). A Norwegian randomised controlled trial with data from 2016 to 2017 indicates that two meals of cod a week during pregnancy improved the iodine status in women with mild-to-moderate iodine deficiency, where the intervention group had significantly higher median UIC (98 μg/L) than the control group (73 μg/L) during the intervention (42). This study also showed large variation in iodine concentration in the individual cod fillets, which can be a challenge when using mean values from a food composition database (42). In our study, lean fish was not a determinant of UIC, which might be explained by low and non-daily intake, and the fact that the UIC mostly reflects what the person has eaten in the past day (43). To affect the median UIC to any extent, quite a few women would have to have eaten lean fish within a few hours before the urine sampling, in contrast to dairy products consumed more regularly.

It is a cause for concern that a significant proportion of the participants in both groups had consumed no dairy products in the past week, while even more participants had not eaten lean fish. Only having a few iodine-rich foods to rely on, coupled with the observed decline in fish and dairy product consumption in Norway since 2015 (23), particularly among young people, makes these groups increasingly vulnerable.

An unexpected finding was that oily fish in the pregnant women was a significant determinant of median UIC. However, calculations from the FFQ reflecting intake during the preceding year revealed no association between oily fish and higher median UIC. This is consistent with the information in the Norwegian Food Composition Table, which shows that oily fish contain relatively low levels of iodine (44), suggesting that the positive association revealed was probably caused by chance.

In our study, the use of iodine supplements led to an elevated UIC concentration and was associated with the largest increase in median UIC. The median UIC of the participants not taking iodine supplements was in fact similar between the pregnant and non-pregnant groups. Pregnant women taking iodine supplements did, however, have adequate iodine status according to the WHO criterion (median UIC of 158 μg/L) (3). This is an improvement from earlier research from MISA that showed an UIC of 112 μg/L (10) in pregnant women taking iodine supplements during the last week, and, thus, our participants had a notably higher UIC. The percentage of pregnant women taking iodine supplements during pregnancy also increased considerably from 18% in the MISA study (10) to 60% in our study, even though pre-pregnancy use remained low.

In individuals who are healthy and consume a varied diet to meet the national requirements, no consistent health benefits have been associated with the consumption of nutrients through supplements (45). However, some groups, such as individuals who are pregnant or planning a pregnancy, or do not consume dairy products or fish, may require dietary supplements (45). Interestingly, results from the MoBa study showed no evidence of a protective effect of iodine supplementation during pregnancy in terms of the child’s risk of language delay, behavioural problems and reduced fine motor skills at 3 years of age (46). The results indicated that a sudden introduction to iodine supplements after conception might negatively affect maternal thyroid function (47). These findings also concur with those of an Italian and a Spanish studies (48, 49). Against this background, it is worrying that fewer than one in four pregnant women in our study used iodine supplements prior to pregnancy. Taking iodine supplements is a good option if women’s diet contains insufficient iodine, but this should be initiated before pregnancy, similar to the recommendations for folic acid (50). In contrast to the 17% use of iodine supplements prior to pregnancy in our study, the data showed that 49% of the pregnant women reported taking folic acid before pregnancy. This widespread use of folic acid suggests that many women are willing to take supplements prior to pregnancy.

The increased UIC observed in the pregnant group compared to the MISA 1 study appears to be a result of a higher percentage of women taking iodine supplements, as discussed. However, one could also consider other causes, such as the updated 2018 Norwegian Antenatal Care Guidelines (50). These guidelines emphasise iodine more than previous documents and offer more detailed recommendations regarding iodine. Consequently, this may have heightened the awareness of healthcare personnel who advise pregnant women. However, our previous research from Northern Norway conducted in 2017/2018, 4 months before the updated guidelines, indicated that healthcare personnel provided little iodine guidance and substantially less than advice on other nutrients. Additional factors such as an increased iodine focus in the Norwegian media or new research could also have contributed to the increase in the use of supplements.

Making appropriate dietary decisions is essential to ensure sufficient iodine intake, and such decisions require knowledge (51). This contrasts greatly with our results, which showed generally poor knowledge and awareness regarding iodine, similar to previous research on Norwegian young women (12, 52). It is concerning that non-pregnant young women know less about iodine than pregnant women do, given the potential implications for near-future pregnancy. The limited knowledge can be seen in relation to our earlier study, which examined the clinical practices of public health nurses and midwives in Northern Norway with regard to ensuring appropriate iodine status during pregnancy. We found that iodine specific recommendations were less commonly given than advice on other nutrients, and that there was insufficient guidance and assessment regarding dietary iodine intake (32).

### Implications

Our findings indicate insufficient iodine status, characterised by a low UIC and low consumption of iodine-rich foods, in addition to limited awareness of the role of iodine as an essential nutrient. We therefore endorse the planned implementation of a Norwegian iodine fortification programme (9, 53) and consider it to be crucial, since it has the potential to increase iodine content in numerous foods. Nonetheless, certain groups within the population may continue to be at risk of deficiency. For instance, women who abstain from consuming bread and bakery products may require alternative strategies, such as targeted iodine supplementation. Consequently, an increased awareness of iodine-rich food sources is needed, including the importance of following the recommendations for milk and dairy products in addition to fish. An increased focus on vulnerable groups, with a special emphasis on non-pregnant women prior to conception, is important, as is a greater effort to raise awareness of healthcare personnel to ensure adequate nutritional support for women of childbearing age.

### Strengths and limitations

A strength of this study is the high number of non-pregnant participants. These were recruited from the general population and students from different places in Northern Norway. Another strength is that our study includes both UIC and iodine intake. A limitation is that most of the pregnant women were recruited from one city and may thus not represent the overall population. Furthermore, the total number of pregnant participants is rather small. A familiar limitation is the use of single-spot urine samples to measure UIC because of the high day-to-day variation of iodine intake; however, the WHO recommends this method at group level, and the variation tends to even out among populations (3). Another limitation is the risk of bias in the FFQ data in the form of over-or-underestimation of different food sources because of self-reporting and because an FFQ does not capture the whole diet. The questionnaire obtaining diet during the last week used in MISA 2 was also limited to sources high in iodine and has not been validated.

## Conclusion

This study has identified inadequate iodine status and insufficient iodine intake among women of childbearing age in Northern Norway. The consumption of iodine-rich foods by the participants was below recommended levels, while the use of iodine supplements was associated with a higher median UIC. Additionally, the findings highlight poor knowledge of the importance of iodine. To ensure adequate iodine status in these vulnerable groups, it is crucial to emphasise the importance of dietary sources of iodine, provide extensive advice on supplement use or implement broader societal interventions.
